# Association between Sexual Activity during Menstruation and Endometriosis: A Case-Control Study

**DOI:** 10.22074/ijfs.2019.5601

**Published:** 2019-07-14

**Authors:** Sanaz Mollazadeh, Behnaz Sadeghzadeh Oskouei, Mahin Kamalifard, Mojgan Mirghafourvand, Nayyereh Aminisani, Mehri Jafari Shobeiri

**Affiliations:** 1 Students’ Research Committee, Nursing and Midwifery Faculty, Tabriz University of Medical Sciences, Tabriz, Iran; 2 Midwifery Department, Tabriz University of Medical Sciences, Tabriz, Iran; 3 Social Determinants of Health Research Center, Tabriz University of Medical Sciences, Tabriz, Iran; 4 Department of Statistics and Epidemiology Faculty of Health Sciences, Tabriz University of Medical Sciences, Tabriz, Iran; 5 Department of Obstetrics and Gynecology, Tabriz University of Medical Sciences, Tabriz, Iran

**Keywords:** Endometriosis, Menstruation, Orgasm, Sexual Activity

## Abstract

**Background:**

The prevalence of endometriosis in the general population is estimated at 7-10%. There are various risk factors for this disease, including early menarche age, prolonged menstruation or no history of pregnancy. It seems that sexual activity leading to orgasm during menstruation increases the retrograde menstruation, sending endometrial tissue to an abnormal sites and thus increasing the risk of endometriosis. The present study is aimed to determine the association between sexual activity during menstruation and endometriosis.

**Materials and Methods:**

This case-control study, conducted in the year 2017, recruited 555 women who were visited at Alzahra Hospital in Tabriz, Northwest of Iran. The case group comprised 185 women of reproductive age with confirmed endometriosis. The control group comprised 370 women of reproductive age without endometriosis visit- ing the hospital for other issues. Data was collected using a researcher-made questionnaire based on previous studies. Bivariate analysis was performed by the chi-squared test and multivariate analysis was done using conditional logistic regression to control confounding variables.

**Results:**

The sexual activity of the two groups during menstruation was significantly different. The occurrence of endometriosis in women who stated they had vaginal intercourse or non-coital sexual activities, leading to orgasm during menstruation, was significantly higher compared to those who stated they did not.

**Conclusion:**

According to our findings, there is an association between sexual activities leading to orgasm during menstruation and endometriosis.

## Introduction

Endometriosis is a benign gynecologic disease (1) defined as the presence of uterine endometrial stroma and glands somewhere other than their natural location (i.e. uterine endometrial cavity). The most frequent places in the pelvic cavity include ovaries, uterosacral ligaments, and the recto-uterine pouch (2). Symptoms of endometriosis are dysmenorrhea, dyspareunia, chronic pelvic pain, irregular menstruation, and/ or infertility (3). Although endometriosis is considered to be a disease of the 21st century, the first references and related symptoms were discovered in ancient Egypt in 1500 BC (4). The prevalence of endometriosis in the general population is estimated at 7-10% (1). Endometriosis is one of the causes of primary and secondary infertility in 30% of women (2).

Endometriosis has a complex and multifactorial etiology (5). The factors involved in the development of endometriosis include hormonal changes (6), genetic changes (7), and changes in the immune system (8). It has been well documented that endometriosis may be present for a long time before it is diagnosed (9). This is especially observed in European countries, as the overall delay in diagnosing the disease is ten years in Austria and Germany, eight years in Spain and UK, seven years in Norway, seven to ten years in Italy and four to five years in Ireland and Belgium (10-1

Although no theory can cover all the manifestations of this disease, the retrograde menstruation is widely accepted to describe the dissemination of endometrial tissue to the peritoneal cavity through open fallopian tubes during menstruation (3, 5). Previous studies report that in 90% of healthy women with open fallopian tubes menstrual blood is present in the peritoneal cavity, as shown by laparoscopy (13). However, it is assumed that the level and volume of retrograde menstruation and the backward movement of endometrial cells have significant effects on the emergence and improvement of endometriosis (14). Studies on uterine pressure during menstruation have shown that myometrium and fallopian tube contraction significantly increase during menstruation and ovulation, respectively, supporting the theory of retrograde menstruation (15). Since this event is regarded as the major etiology for the improvement of endometriosis (9), it is essential to identify factors, which block menstrual blow flow and facilitate the retrograde movement of menstrual blood (16).

It is hypothesized that sexual activity leading to orgasm during menstruation may increase retrograde menstruation, seeding endometrial tissue in other locations, and thus increasing the risk of endometriosis. So far, few studies have examined the relationship between sexual activity during menstruation and endometriosis (16-18). Based on a case-control study in the Yale University School of Medicine, on the relationship between prevalence of endometriosis and sexual behaviors leading to orgasm during menstruation, the tendency to perform such activities during menstruation was lower in women with endometriosis compared to women without endometriosis (17). The results of another study at the University of Pennsylvania, Philadelphia, on the relationship among sexual activity during menstruation, endometriosis and pelvic inflammatory disease showed that endometriosis was higher in women who had sexual intercourse during menstruation compared to those who stated they did not (16). Based on the noted contradictory results and the need for further research, the aim of the present study is to answer the question of whether sexual activity leading to orgasm during menstruation can be a risk factor for occurrence of endometriosis. 

## Materials and Methods

### Study type and participants

This case-control study, which was done in 2017, recruited women at reproductive age (20-50 years), with or without endometriosis. The participants in the case group were selected from women with endometriosis visiting Alzahra Hospital over the past two years, who had undergone laparoscopy and open surgery with a histological diagnosis of endometriosis. Participants in the control group were selected from the same age group of women visiting the same hospital for other reasons including vaginitis and an annual checkup. The absence of endometriosis in the control group was confirmed by a gynecologist colleague based on their signs and symptoms. The final selections were done based on our inclusion and exclusion criteria. The study inclusion criteria were: i. Age of 20-50 years, ii. Diagnosis of endometriosis by open surgery or laparoscopy and histologic diagnosis of endometriosis or the presence of endometrioma (case group), iii. Being married, iv. Being Iranian, v. Willingness to participate, vi. Absence of endometriosis (control group), vii. No history of tubectomy (control group), and viii. No history of infertility (control group). 

The study exclusion criteria were: i. Being menopausal (amenorrhea for over a year), ii. Being suspected of endometriosis or endometrioma (control group), iii. Having endometriosis in the surgical site or involvement of remote organs, e.g. lungs or brain, iv. Having breast, ovarian, or endometrial cancer, v. Having polycystic ovarian syndrome (PCOS), vi. Having any other life-threatening disease, and vii. Suffering from chronic pelvic pain. 

In this study, the sample size was determined according to the results of a pilot study on 150 participants and considering an odds ratio (odds of having sexual activity in the menstruation in the case group compared with the control group) to be about 1.8, was determined as n1=185 (case group), n2=370 (control group), and n=555 (total) (with the case-to-control ratio of 1:2). 

### Data collection

The present study was confirmed by the Ethics Council of Tabriz University of Medical Sciences (ethics code: 5/ D1003687). Afterward, data collection was started in Alzahra Hospital, Tabriz, which is a referral Gynecology and Midwifery Hospital in Northwest of Iran. We reviewed pathological results in the medical files that were available at Alzahra Hospital and registered women patients with endometriosis, as confirmed by histological diagnosis through laparoscopy or open surgery, for our study. The addresses and phone numbers of all considering patients, who had been identified over the past two years, were extracted from their records. They were contacted by telephone, the research objectives and methods were briefly explained to them, the study inclusion and exclusion criteria were checked, and finally they were invited to participate in the study. For those who were willing to take part, questionnaires were filled in through the interview. For the patients’ comfort, the interviewer and the patients were of the same gender. After sampling was done in the case group, the members of the control group were selected through purposive sampling from those visiting the gynecology clinic of the same center for other issues, such as vaginitis or an annual visit, and did not have endometriosis, as diagnosed based on symptoms by a gynecologist colleague. Research objectives and methods were first explained to them. For those who were willing to participate, inclusion and exclusion criteria were checked, and in case they met the criteria, they were recruited and questionnaires were completed by the researcher through interviews. Informed consent forms were obtained from all participants, and those in both case and control groups were matched for age ± 2 years. 

### Data collection tools

Data were collected by the researchers through interviews and using researcher-made questionnaires based on previous studies, highlighting sociodemographic and sexual activity characteristics. The sociodemographic characteristics questionnaire included questions on age; the level of education, employment, the level of income, smoking, alcohol use, the history of any diseases, allergies, and endometriosis in first-degree relatives (mother, sisters, and aunts). The sexual activity and reproductive and menstruation characteristics questionnaire included questions on vaginal or non-coital sexual activity (by touching other body parts by the person or her spouse to achieve sexual pleasure) and anal intercourse, leading to orgasm during menstruation, cycle length, cycle intervals, number of pregnancies, menarche age, age at first pregnancy, oral contraceptive pill (OCP) user, intrauterine device (IUD) user, dysmenorrhea, dyspareunia, and recurrent vaginitis. Content and face validity were used to confirm the validity of the questionnaires, they were given to 10 faculty members and corrections were applied based on their opinions. 

### Data analysis 

Data were analyzed in SPSS 21 software. Sociodemographic and sexual activity characteristics during menstruation were described using descriptive statistics including frequency (percentage). Sociodemographic characteristics were compared between the two groups using chi-squared test, chi-squared test for trend, independent samples t test, and Fisher’s exact test. To determine the relationship between sexual activity during menstruation and endometriosis, chi-squared test was performed in the bivariate analysis. Conditional logistic regression was employed in the multivariate analysis to control confounding variables (level of education, level of income, occupation, cycle length, cycle interval, number of pregnancies, menarche age, age at first pregnancy, OCP user, IUD user, dysmenorrhea, dyspareunia and recurrent vaginitis). Because no woman in the control group reported a history of this disease in her first-degree relatives, the family history was not included in the multivariate regression as a confounding factor. In this analysis, the odds ratio and confidence interval was set at 95%, and P<0.05 was considered significant. 

## Results

In this study, 185 women with endometriosis and 370 women without endometriosis were analyzed. The participants’ mean age was 35.21 years (SD: 7.09) in the case group and 35.28 years (SD: 7.03) in the control group. The two groups significantly were differed regarding the level of education; the percentage of participants with academic degrees in the case group was twice as high as those in the control group (P<0.001). Moreover, 32 (17.3%) women of the case group and 10 (2.7%) women of the control group were employed, again indicating a significant difference between the two groups (P<0.001). However, the two groups were similar regarding the sufficiency of monthly income (P=0.698). The two groups were compared in a history of diseases such as diabetes, hypothyroidism, hypertension, cardiovascular diseases, cerebrovascular diseases, seizures and asthma, and did not show any significant differences (P=0.860). The two groups were also similar regarding an autoimmune disease history, e.g. rheumatoid arthritis, multiple sclerosis, and lupus erythematosus (P=0.669). There were 38 (20.5%) women in the case group and 47 (12.7%) women in the control group with a history of allergies, indicating a significant difference between the two groups (P=0.016). Nevertheless, both groups were similar regarding the type of allergies (seasonal, food, drug, or skin) (P=0.946). In the case group 13 (7%) women reported a history of endometriosis in their mothers and sisters, and 7 (3.8%) women reported this in their aunts, while no woman in the control group reported a history of this disease in her first-degree relatives, demonstrating a significant difference between the groups (P<0.001). Only one woman in the control group had a history of smoking, and no one in either group had a history of alcohol use (Table 1). 

**Table 1:** Comparison of sociodemographic characteristics in case and control groups


Social-demographic characteristic	Case n=185	Control n=370	P value

Age (Y)	35.21 (7.09)^§^	35.28 (7.03)^§^	0.909^*^^*^
Education			<0.001^‡^
Illiterate/Primary	57 (30.8)	129 (34.9)	
Guidance/High school	37 (20.0)	82 (22.2)	
Diploma	39 (21.1)	107 (28.9)	
Academic	52 (28.1)	52 (14.1)	
Occupation			<0.001^*^
Housewife	153 (82.7)	360 (97.3)	
Employed	32 (17.3)	10 (2.7)	
Job type			1.000^†^
Doctor/University professor	7 (21.9)	2 (20.0)	
Employee	21 (65.6)	7 (70.0)	
Free	4 (12.5)	1 (10.0)	
Adequacy of monthly income			0.698^‡^
Weak	42 (22.7)	79 (21.4)	
Average	102 (55.1)	197 (53.2)	
Good/Very good	41 (22.2)	94 (25.4)	
Having a history of a disease	24 (13.0)	50 (13.5)	0.860^*^
Having a history of autoimmune disease	1 (0.5)	4 (1.1)	0.669^†^
Having a history of allergies	38 (20.5)	47 (12.7)	0.016^*^
The history of first-degree relatives			<0.001^†^
Yes	20 (10.8)	0	
No	165 (89.2)	370 (100.0)	


Data are presented n (%). *; Chi-squared test, ‡; Chi-squared test for trend, †; Fisher’s exact test, §; Mean ±SD, and **; Independent samples t test. Only one woman in the control group had a history of smoking, and no one in either group had a history of alcohol use.

Regarding vaginal intercourse during menstruation, the two groups were compared using multivariate logistic regression, while controlling the effects of possible confounding variables, such as the level of education, income, occupation, cycle length, cycle interval, number of pregnancies, menarche age, age at first pregnancy, OCP user, IUD user, dysmenorrhea, dyspareunia and recurrent vaginitis. The results showed that the risk of endometriosis approximately was five times higher in those women who stated they had vaginal intercourse during menstruation compared to those who stated they did not [(P<0.001), odds ratio (OR) (95% confidence interval (CI)=5.23 (2.16 to 12.66)]. Furthermore, 6 (20%) participants in the case group and 1 (3.6%) participant in the control group reported that they always had vaginal intercourse during menstruation, demonstrating a significant difference between the groups (P<0.001). Both groups were similar with regard to the days of vaginal intercourse (first three days, second 3 days, all days of menstruation) (P=0.111). Moreover, the risk of endometriosis was approximately three times higher in those women who stated they had non-coital sexual activity during menstruation compared to those who stated they did not [(P=0.010), OR (95% CI)=2.90 (1.28 to 6.55)]. In addition, 9 (23.7%) participants in the case group and 6 (14.6%) participants in the control group reported that they always had non-coital sexual activity during menstruation, indicating no significant difference between the two groups based on a chi- squared test (P=0.141). Moreover, 2 (1.1%) participants in the case group and 15 (4.1%) participants in the control group stated that they have anal intercourse during menstruation, but there was no significant difference between the two groups [(P=0.130), OR (95% CI) = 0.08 (0.03 to 2.09)] (Tables2, 3). 

**Table 2:** Comparison of sexual activity during menstruation and reproductive and menstruation characteristics in case and control groups based on bivariate test


Sexual activity during menstruation and reproductive and menstruation characteristics	Case n=185	Control n=370	P value

Vaginal sex activity			0.002^*^
Yes	30 (16.2)	28 (7.6)	
No	155 (83.8)	342 (92.4)	
Sexual activity without vaginal penetration			0.003^*^
Yes	38 (20.5)	41 (11.1)	
No	147 (79.5)	329 (88.9)	
Anal sex activity			0.075^†^
Yes	2 (1.1)	15 (4.1)	
No	183 (98.9)	355 (95.9)	
Age at menarche			<0.001^*^
≤ 12	54 (29.2)	64 (17.3)	
>12	131 (70.8)	306 (82.7)	
Cycle interval			0.012^*^
≤ 28	109 (58.9) 176 (47.6)		
> 28	76 (41.1)	194 (52.4)	
Cycle length			<0.001^*^
≤ 7	136 (73.5)	357 (96.5)	
> 7	49 (26.5)	13 (3.5)	
Pregnancy number			<0.001^*^
0/1	102 (55.1)	80 (21.6)	
≥ 2	83 (44.9) 290 (78.4)		
OCP user			0.303^*^
Yes	75 (40.5)	167 (45.1)	
IUD user			0.017^*^
Yes	42 (22.7)	120 (32.4)	
Age at first pregnancy			0.037^*^
≤ 20	43 (30.1)	146 (40.0)	
> 20	100 (69.9)	219 (60.0)	
Dysmenorrhea			<0.001^*^
Yes	136 (73.5)	43 (11.6)	
Dyspareunia			<0.001^*^
Yes	82 (44.3)	7 (1.9)	
Recurrent vaginitis			<0.001^*^
Yes	50 (27.0)	18 (4.9)	


*; Chi-squared test and †; Fisher’s exact test.

**Table 3:** Comparison of sexual activity during menstruation in case and control groups based on bivariate and multivariate logistic regression


Variable	Unadjusted		Adjusted	
	P value	OR (CI 95%)	P value	OR (CI 95%)

Vaginal sex activity	0.002	2.36 (1.36 to 4.09)	<0.001	5.23 (2.16 to 12.66)
Sexual activity without vaginal penetration	0.003	2.07(1.28 to 3.36)	0.010	2.90 (1.28 to 6.55)
Anal sex activity	0.075	0.25(0.05 to 1.14)	0.130	0.08 (0.03 to 2.09)


Conditional logistic regression was employed (P<0.1) in the multivariate analysis to control confounding variables: level of education, level of income, occupation, cycle length, cycle interval, pregnancy number, menarche age, age at first pregnancy, OCP user, IUD user, dysmenorrhea, dyspareunia, and recurrent vaginitis. CI; Confidence interval, OR; Odds ratio, OCP; Oral contraceptive pill, and IUD; Intrauterine device.

## Discussion

The present study is the first study in Iran, which examined the association between sexual activity during menstruation and endometriosis. Our results revealed that vaginal intercourse and non-coital sexual activity leading to orgasm during menstruation increase the risk of endometriosis.

The case and control groups were significantly different regarding the level of education and occupation. Most recent epidemiological studies on risk factors for endometriosis have shown an increased incidence of the disease among women of high socioeconomic and occupational status (19). Results of the present study are consistent with the noted results. One possible justification for this relationship may be attributed to the diagnostic bias, because women of high socioeconomic status may have more awareness of their health-related issues (19-21). A strong evidence that shows the importance of a family history of endometriosis among women with the disease, is occurrence of endometriosis in twins (22, 23). In the present study, the participants in the case group reported a family history of endometriosis in their first-degree relatives, while no women in the control group reported a history of this disease in her first-degree relatives, therefore, family history was not employed into the model as a confounding variable.

There are few studies on the association between sexual activity during menstruation and endometriosis. For instance, Meaddough et al. (17) in the US explored the effect of sexual activity, orgasm, and health-related behaviors during menstruation on endometriosis. Results demonstrated that women with endometriosis were less willing to have repeated or occasional sexual activity during menstruation compared to those without endometriosis, a difference which turned out to be statistically significant. Moreover, in their study the case group reported less sexual activity leading to orgasm during menstruation than the control group, showing a significant difference between the two groups. Based on Meaddough’s inference a possible explanation for these results could be the limitation of the research instrument or the presence of confounding variables such as dyspareunia, which was not included in the questionnaire. Dyspareunia may lead to an unwillingness in women with endometriosis to have sex, thereby making them report a lower willingness than the control group. Furthermore, Meaddough concluded that sexual activity or orgasm during menstruation might facilitate the blood flow through the cervix (17). Other studies have shown that uterine contractions and pressure are increased during menstruation (16). Since sexual activity and orgasm during menstruation appear to increase uterine contractions as well, this type of activity may in fact lead to retrograde menstruation, which is the major etiology of endometriosis. In Meaddough’s study, the number of women with and without endometriosis was determined based on self-report and not based on specialized criteria. Furthermore, questionnaires were sent and completed by the participants via email. Therefore, this study recommended further detailed studies on the effect of sexual activity during menstruation on the development of endometriosis (17). On the other hand, in the present study, women with endometriosis were selected based on histological diagnosis through laparoscopy, and those without endometriosis were selected based on signs and symptoms, inclusion and exclusion criteria, and confirmation of the absence of endometriosis by a gynecologist. Moreover, questionnaires were completed by the same interviewer for both groups, and all confounding variables were controlled as much as possible. To this end, the two groups were matched for age, and all possible confounding variables were controlled in statistical analyses. 

Another study was conducted by Filer and Wu (16) in the University of Pennsylvania, Philadelphia to investigate the effect of sexual activity on endometriosis and pelvic inflammatory disease. The definite diagnosis of endometriosis and tubal factor infertility was done by laparoscopy or laparotomy. Subjects were asked about their history of sexual activity during menstruation and history of the pelvic inflammatory disease. The results showed that the prevalence of endometriosis was higher in women who tended to have repeated or occasional sexual activity during menstruation compared to those who did not. The prevalence of endometriosis was (17.5%) in women who had repeated or occasional sexual activity, and (10.9%) in those who rarely had sexual activity during menstruation, demonstrating a significant difference between the two groups. However, the groups were similar regarding pelvic inflammatory disease. Another study was conducted by Samir et al. (18) in Doha, Qatar, to examine sexual activity during menstruation as a predisposing factor for endometriosis. First, participants were asked about sexual activity during menstruation. A total of 78 participants were divided into two groups: the first group: 51 participants with a sexual activity history during menstruation and the second group: 27 women without a history of sexual activity during menstruation. Then, abdominal ultrasound, transvaginal ultrasound, or both were performed before laparoscopy or open surgery. Their results revealed that in the first group: 36 (66%) women tested positive for endometriosis and the second group: 9 (34%) were negative for endometriosis, showing a significant difference between the presence and absence of sexual activity leading to orgasm during menstruation and endometriosis (18). The results of the present study are consistent with the results of Samir’s group. Based on our findings, the risk of endometriosis is approximately five times higher in women who stated they had vaginal intercourse leading to orgasm during menstruation and three times higher in those with non-coital sexual activity leading to orgasm during menstruation, compared to those who stated they did not.

In this study, there was no significant difference between the groups in terms of having anal intercourse leading to orgasm during menstruation. However, due to the limited number (only two women in the control group), a complete conclusion is not possible.

In this study, an attempt was made to select women with and without endometriosis based on precise medical diagnosis. Furthermore, the most important and relevant factors with endometriosis were examined while controlling confounding variables. 

In this study, validity was only confirmed through face and content validity qualitatively and the quantitative indices such as content validity index (CVI) and content validity ratio (CVR) weren’t calculated. Also, considering the criterion for the definite diagnosis of endometriosis is histologic diagnosis through laparoscopy or laparotomy, one of the other shortcoming of this study is that the control group wasn’t selected based on histologic diagnosis. Thus, future studies should be conducted on selected case and control participants from women, in whom the presence or absence of endometriosis is confirmed by laparoscopy or laparotomy. 

## Conclusion

Based on the results of the present study, vaginal intercourse or non-coital sexual activity leading to orgasm during menstruation increases the risk of endometriosis in women during reproductive age. This study has raised interesting issues and requires further investigation to better understand the mechanism of occurrence of endometriosis in such cases.
